# Glances at the Hospitals

**Published:** 1901-10-12

**Authors:** 


### GLANCES AT THE HOSPITALS.

### THE SOUTH DEVON AND EAST CORNWALL HOSPITAL.

At this hospital there was a deficit on the year's working of £263. The total ordinary income amounted to £6,491, and the total expenditure to £6,755. A handsome donation of £2,000 was received from Mrs. Cubitt, of Kingsbridge. At the end of the year 94 patients remained in the hospital, and 1,188 were admitted during the year. Of these 674 were cured, 40 greatly relieved, 296 relieved, 15 were made out-patients, 60 unrelieved or left at their own request, 3 were discharged to fever hospital, 100 died, and 94 remained under treatment. The surgical cases numbered 1,074, and the medical cases 208. The report of the operations performed during the year shows that there were 550 major operations, and the sex, age, nature of operation, result, and remarks are given in every case. Any group of operations can be easily referred to, and the table is most carefully prepared. The anæsthetic table shows that chloroform was given 626 times, a mixture of chloroform and ether 127 times, nitrous oxide and ether 49 times, ether alone 16 times, and the A.C.E. mixture 9 times. The purely medical statistics are very meagre. The numbers of patients treated for the various diseases are given, but no results are appended. The 208 cases may all have recovered or all died for anything that appears on the table.

### THE GLASGOW EYE INFIRMARY.

The ordinary income was £4,285, and the ordinary expenditure was £4,207, showing a balance to the good of £78. If it be granted that each out-patient cost 1s. 6d., the average cost per bed occupied in the hospital was £31 15s. and the average cost of each case treated in the house was £1 16s. 7d. The total number of patients treated during the year was 12,696, but 1,213 of these remained under treatment from the previous year. Of the total number 9,164 were cured, 2,069 were relieved, 175 were advised, 194 were deemed incurable, and 1,094 remained under treatment. The tables show the numbers treated in the various groups of diseases, but no results are given, without which the statistics are shorn of nearly all their value. There were 151 cases of iritis, but we learn nothing about these further than that 58 were specific, 61 rheumatic, 19 serous, and 23 traumatic. Optic neuritis figures 53 times, and the operation for cataract was performed 225 times, with what results we know not. A special report is presented by the pathologist. During the year 127 eyes and 10 tumours were examined. In 16 cases the eye had been injured by broken glass, in 3 cases by fire-arms, in 9 cases by blows from a fist, and it is curious to note that in 3 cases the eye was destroyed by a penetrating wound received from a fork when being used to untie a boot-lace. The museum was enriched by 52 mounted specimens and 100 microscopic preparations. Nearly 100 medical students attended the hospital.

